# Lipoamide Acts as an Indirect Antioxidant by Simultaneously Stimulating Mitochondrial Biogenesis and Phase II Antioxidant Enzyme Systems in ARPE-19 Cells

**DOI:** 10.1371/journal.pone.0128502

**Published:** 2015-06-01

**Authors:** Lin Zhao, Zhongbo Liu, Haiqun Jia, Zhihui Feng, Jiankang Liu, Xuesen Li

**Affiliations:** 1 Center for Mitochondrial Biology and Medicine, The Key Laboratory of Biomedical Information Engineering of Ministry of Education, School of Life Science and Technology and Frontier Institute of Science and Technology, Xi’an Jiaotong University, Xi’an, China; 2 Department of Basic Science and Craniofacial Biology, College of Dentistry, New York University, New York, NY, United States of America; 3 California Institute for Biomedical Research, La Jolla, CA, United States of America; 4 Tianjin Key Laboratory of Exercise Physiology and Sports Medicine, Tianjin University of Sport, Tianjin, China; 5 Institute for Cancer Medicine, Luzhou Medical College, Luzhou, Sichuan, China; Indiana University College of Medicine, UNITED STATES

## Abstract

In our previous study, we found that pretreatment with lipoamide (LM) more effectively than alpha-lipoic acid (LA) protected retinal pigment epithelial (RPE) cells from the acrolein-induced damage. However, the reasons and mechanisms for the greater effect of LM than LA are unclear. We hypothesize that LM, rather than the more direct antioxidant LA, may act more as an indirect antioxidant. In the present study, we treated ARPE-19 cells with LA and LM and compared their effects on activation of mitochondrial biogenesis and induction of phase II enzyme systems. It is found that LM is more effective than LA on increasing mitochondrial biogenesis and inducing the expression of nuclear factor erythroid 2-related factor 2 (Nrf2) and its translocation to the nucleus, leading to an increase in expression or activity of phase II antioxidant enzymes (NQO-1, GST, GCL, catalase and Cu/Zn SOD). Further study demonstrated that mitochondrial biogenesis and phase II enzyme induction are closely coupled via energy requirements. These results suggest that LM, compared with the direct antioxidant LA, plays its protective effect on oxidative damage more as an indirect antioxidant to simultaneously stimulate mitochondrial biogenesis and induction of phase II antioxidant enzymes.

## Introduction

The dysfunction of retinal pigment epithelium (RPE) is strongly related to age-related macular degeneration (AMD) [1, 2]. Oxidative stress has been proven as a key factor in AMD pathology by a number of studies and RPE is very susceptible to oxidative stress [3]. Since mitochondria are the main producing site and also the most susceptible target of reactive oxygen species (ROS), it is important to study both mitochondria and antioxidant systems in RPE.

Mitochondria, which provide most of the needed energy for the cell, are vital for normal cell physiology and health. Mitochondrial dysfunction has been proven to be related to aging [4] and to a variety of diseases [5], including type II diabetes [6] and neurodegenerative diseases [7]. Mitochondrial function is related to mitochondrial content [8], and mitochondrial biogenesis is necessary for keeping proper mitochondrial mass and function. Studies indicate that mitochondrial biogenesis declines in type II diabetes [9, 10] and aging; drugs that enhance mitochondrial biogenesis can promote fatty acid oxidation and improve insulin resistance [11]; calorie restriction, the most effective anti-aging strategy, promotes mitochondrial biogenesis [12], which may play an important role in the anti-aging process [13].

The key transcriptional coactivator in mitochondrial biogenesis is peroxisome proliferator-activated receptor coactivator 1 alpha (Ppargc1a) [14], which regulates downstream mitochondrial biogenesis-related transcription factors, including nuclear respiration factors 1 and 2 (NRF1 and NRF2), and mitochondrial transcription factor A (Tfam) [15]. These transcription factors activate expression of mitochondrial component proteins, such as the electron transfer chain subunits [15].

Intracellular antioxidant systems are important for the cell to counteract oxidative stress. The most important endogenous antioxidant system involves nuclear factor erythroid 2-related factor 2 (Nrf2) induced phase II antioxidant enzymes [16], including NADPH-quinone-oxidoreductase 1 (NQO-1), glutathione S-transferase (GST), γ-glutamyl-cysteinyl-ligase (GCL), catalase, and Cu/Zn SOD. All of these enzymes have antioxidant response elements (ARE) in their promoter region; the heterodimer of Nrf2 and small Maf binds to the ARE and activates gene transcription. Nrf2 is inactive when confined to the cytoplasm, but can be translocated to the nucleus in the presence of appropriate inducers or environmental conditions [17].

(R)-α-lipoic acid (LA) has been widely investigated as an antioxidant and used to prevent and treat neuropathy of type II diabetes and neurodegenerative diseases [18–20]. LA also demonstrated protective effect on both primary human fetal retinal pigment epithelium cells and ARPE-19 cells in oxidative stress conditions[21]. However, lipoamide (LM), the neutral amide of LA, has been studied inadequately. In a previous study [22], we found pretreatment with LM was more potent than with LA in protecting ARPE-19 cells from acrolein-induced mitochondrial dysfunction and oxidative damage. Acrolein, a main toxin in cigarette smoking and air pollution, is an active chemical that reacts immediately with biological molecules; therefore, if the cells were to successfully confront acrolein-induced stress and survive, beneficial changes induced by the pretreatment must occurred [21–23]. However, the mechanisms underlying the more potent effect of LM than LA remain unclear. Antioxidants can directly react with reactive oxygen species or induce cellular antioxidant system behaving as indirect antioxidants. In this study, we examined the indirect antioxidant effects of LM, compared with LA, on stimulation of mitochondrial biogenesis and induction of the phase II enzyme system, and found that LM might be a more effective indirect antioxidant than LA.

## Materials and Methods

### Materials

DL-α-LM was purchased from Sigma Chemical Co. (St. Louis, MO). (R)-α-LA tris salt was a gift from Klaus Wessel, Viatris, Germany. Fetal bovine serum was from Hyclone (Logan, UT). 2',7'-Dichlorodihydrofluorescein diacetate (H_2_DCFDA), 5,5’,6,6’-tetrachloro-1,1’,3,3’-tetraethylbenzimidazolyl-carbocyanine iodide (JC-1), and MitoTracker Green were purchased from Molecular Probes (Eugene, OR). The BCA protein assay reagent kit was from Pierce (Rockford, IL). Polyclonal rabbit antibodies against PPARGC1a and Nrf2 were purchased from Santa Cruz Biotechnology Inc. (San Diego, CA); monoclonal antibodies for Complex I (NADH ubiquinone oxidoreductase 39-kDa subunit), Complex II (succinate-ubiquinone oxidoreductase 70-kDa subunit), Complex III (ubiquinol–cytochrome c oxidoreductase core II 50-kDa), Complex IV (cytochrome c oxidase 48-kDa) and Complex V (ATP synthase, 53–kDa), were from Molecular Probes (Eugene, OR); monoclonal antibodies for GCLc, catalase and GST were from Stressgen Biotechnologies Corporation (Victoria, British Columbia, Canada). TRIzol, primers and other reagents for cell culture were from Invitrogen (Carlsbad, USA). 2,3-Naphthalenedicarboxyaldehyde, buthionine sulfoximine (BSO), diethyl maleate and other common reagents were purchased from Sigma Chemical Co. (St. Louis, MO).

### Cell culture and treatments

The human ARPE-19 cell line was obtained from Nancy J. Philp (Thomas Jefferson University, Philadelphia, PA) and was cultured according to her methods. ARPE-19 cells were cultured in DMEM-F12 medium supplemented with 10% fetal bovine serum, 2 mmol/L L-glutamine, 100 U/ml penicillin and 100 μg/ml streptomycin. ARPE-19 cells were used within 10 generations. For all experiments, if not otherwise stated, cells were cultured to 60% density then treated with LM or LA for 48 h, and the cell density would grow to 90%-95% confluence. Then the cells were washed with PBS and collected for various assays.

### Mitotracker staining for viable mitochondria

Cells were cultured in six-well plates. After LM treatment, cells were stained with 100 nmol/L Mitotracker Green in medium for 30 min; then the staining medium was discarded after centrifugation. Cells were resuspended with PBS, and fluorescence was detected with BD FACS Arisa [24].

### Mitochondrial membrane potential (MMP) assay

MMP changes in live ARPE-19 cells cultured in 96-well plates were determined by using the lipophilic cationic probe JC-1 [25] with a fluorescence spectrometer (Flex StationII 384, Molecular Devices). The fluorescence ratio (590 nm emission to 530 nm emission) was used for quantitative analysis.

### Reactive oxygen species (ROS) assay

ROS generation was detected using H_2_DCFDA [26]. Fluorescence was determined by a fluorescence spectrometer (Flex StationII 384, Molecular Devices) at 488 nm (excitation) and 525 nm (emission).

### Intracellular adenosine 5′-triphosphate (ATP)

Cells were cultured in six-well plates. After various treatments, cells were lysed by 0.5% Triton X-100 in 100 mM glycine buffer, pH 7.4. Intracellular ATP levels were assayed with an ATP bio-luminescence assay kit (Sigma) based on the luciferase-catalyzed oxidation of d-luciferin.

### Oxygen consumption determination

Oxygen consumption was determined using the BD Oxygen Biosensor System (BD Biosciences). Generally, cells were cultured in 6-well plates, after indicated treatment cells were suspended in culture medium and subsequently transferred to the 96-well oxygen biosensor plate. The plate was tightly sealed to isolate outside atmosphere. The oxygen was consumed by the cells and the oxygen-sensitive fluorescence dye could emit fluorescence under excitation light. Levels of oxygen consumption were measured under baseline conditions. Fluorescence was recorded using a fluorescence microplate reader (Flex StationII 384, Molecular Devices) at 1-min intervals for 2 h at an excitation of 485 nm and emission of 630 nm [27–29]. Cell numbers were counted with a hemocytometer. The maximum slope of fluorescence (in fluorescence units/s) was measured and converted into percent of control (set to 100%).

### Real time PCR for mitochondrial DNA quantification and mRNA expression assay

Total DNA was isolated by a standard method with a DNA isolation kit (U-gene Biotech Co. Ltd, China). Total RNA was isolated with Trizol following a standard protocol. cDNA was obtained with Reverse Transcriptase from Toyobo Co. Ltd (Osaka, Japan) and oligo dT primers from Takara Co. (Dalian, China). Real-time PCR quantification was preformed using Master SYBR Green Premix and expressed relative to 18S rDNA or 18S rRNA level in the same sample [30]. For mitochondrial DNA copy numbers, D-LOOP region of the mitochondrial genome was selected for quantification and primers were designed as shown in Table 1. The ratio of D-LOOP to 18S rDNA was caculated as relative mitochondrial DNA copy number. Primers of Mn superoxide dismutase (MnSOD), Cu/ZnSOD, thioredoxin 2 (Trx 2), peroxiredoxin 3 (Prx 3), and peroxiredoxin 5 (Prx 5) were the same as described by Valle et al. [31]. The specificity of the primers was examined by both agarose gel electrophoresis and dissociation curve generation. All the primers and annealing conditions are shown in Table 1. All PCR amplification was performed using the Mx3000P system (Applied Biosystems, United States). Data were calculated using the formula 2^-ΔCt^, where ΔCt = Ct_target_-Ct_18s_ [32].

**Table 1:** Primers and annealing temperature.

gene	Annealing Temp.	Forward	Reverse
18sRNA	55	CATTCGAACGTCTGCCCTATC	CCTGCTGCCTTCCTTGGA
D-LOOP	55	CCCACTAGGATACCAACAAAC	CCAGATGTCGGATACAGTTCA
TFAm	60	ATTCCAAGAAGCTAAGGGTG	CTTCCCAAGACTTCATTTC
NRF1	65	GCCGTCGGAGCACTTACT	CTGTTCCAATGTCACCACC
NRF2alpha	60	GATTTTTTTCAGCGGGTTC	ACTGCCATAGTTGGATTTG
MnSOD	55	AGGTTAGATTTAGCCTTATTCCAC	TTACTTTTTGCAAGCCATGTATCTTTC
Prx 3	60	CCTTTGGATTTCACCTTTGTGTG	CAAACCACCATTCTTTCTTGGTG
Prx5	60	CCAATCAAGGTGGGAGATGCC	GCAGGTGTGTCTTGGAACATC
TRX2	60	GTCCACACCACTGTGCGTGG	TTGCAGGGAGATGGCTCAGCG
UCP-2	55	TACAAAGCCGGATTCCGGCAGC	CTCCTTGGATCTGTAACCGGAC
Cu/ZnSOD	55	CGGAGGCTTTGAAGGTGTGG	CTCCAACATGCCTCTCTTCATCC

### Western blots for protein expression

Total protein or nuclear protein was isolated with a commercial lysis buffer (Pierce, Thermo Scientific) following standard instructions. Protein was frozen at -20°C before use. Protein concentrations were quantified by BCA assays (Pierce BCA Protein Assay Kit, Thermo Scientific), and samples were adjusted to the same concentration with lysis buffer. For each sample, the same quantity of protein (10 to 30 μg) was subjected to 10% or 12% SDS-PAGE, then proteins separated on the gel were transferred to a nitrocellulose membrane and blocked with 5% defatted milk/TBST for 1 h at room temperature. The membrane was incubated with primary antibodies at 4°C overnight. The primary antibodies were diluted in 5% defatted milk/TBST with specific dilutions as follows: Nrf2 (1:1000), PPARGC1a (1:1000), complex I (1:2000), complex II (1:4000), complex III (1:4000), complex IV (1:1000), complex V (1:4000), catalase (1:1000), GCLc (1:1000), NQO-1 (1:2000), GST(1:500), β-actin (1:10000), and α-tubulin (1:10000). After washing membranes with TBST three times, membranes were incubated with horseradish peroxidase-conjugated antibody for 1 h at room temperature. Western blots were developed using ECL (Roche Manheim, Germany) and quantitative analysis based on optical density was performed with Quantity One software.

### Catalase activity assay

Catalase activity was measured with a commercial kit (Nanjing Jiancheng Bioengineering Institute, Nanjing, China). Generally, cells were lysed with lysis buffer and centrifuged at 13,000 *g* for 15 min at 4°C; the supernatant was collected for the test. The reaction system contained 50 mmol/L hydrogen peroxide; 5 min after adding 15 μg protein, the reaction was terminated; the remaining hydrogen peroxide was oxidized by peroxidase to N-4-(antipyryl)-3-chloro-5-sulfonate-benzoquinonemonoimine. The red color produced with an absorbance maximum at 520 nm was measured with a spectrometer.

### NQO-1 activity assay

NQO-1 activity was measured as previously described [33] with minor modifications. Cells were washed three times with PBS and scraped from the dishes using a rubber, and then collected in TE buffer (20 mmol/L Tris HCl containing 2 mmol/L EDTA, pH 7.4). This was followed by sonic disruption on ice, 4 x 5 s with 10 s interval between sonications; after centrifugation at 13,000 *g* the supernatant was used for further testing. The reaction mixture (200 μl final volume) consisted of 25 mmol/L Tris–HCl (pH 7.4), 80 μmol/L 2,6-dichlorophenolindophenol (DCPIP), 0.2 mg/ml BSA and 0.01% (v/v) Tween-20, with or without 10 μmol/L dicoumarol, and 10 μg cell lysate protein. The reaction was started by the addition of 180 μmol/L NADPH. Reduction of DCPIP was measured at room temperature for 1–2 min at 600 nm (ε = 21×10^3^M^−1^ cm^−1^). NQO1 activity was considered to be the dicoumarol-inhibitable part of DCPIP reduction.

### GST activity assay

Cells were lysed ultrasonically in 10 mmol/L sodium phosphate buffer, pH 6.5. The protein content of the cell lysate was quantified by the BCA method. GST activity was measured using 5 mg protein, 1 mM GSH, 1 mM chloro-2, 4-dinitrobenzene, 3 mg/ml BSA in 10 mM sodium phosphate buffer. The mixture was scanned at 340 nm for 5 min at 25°C [34].

### GCL activity and GSH level assays

The methods used to assay GCL activity and GSH levels were as previously described [35]. Briefly, a fluorescent dye 2,3-naphthalenedicarboxaldehyde (NDA), reacts with both glutamylcysteine (GC) and GSH to form NDA–c-GC or NDA–GSH. For each sample, two tubes were prepared, one for the GSH test, the other for the GCL activity test. In both tubes, 50 μl GCL reaction cocktail (400 mmol/L Tris, 40 mmol/L ATP, 20 mmol/L L-glutamic acid, 2.0 mmol/L EDTA, 20 mmol/L sodium borate, 2 mmol/L serine, 40 mmol/L MgCl_2_) was pre-incubated with 50 μl cell lysate (2000 μg/ml) for 5 min at 37°C; then the GCL reaction was initiated by adding 50 μl of 2 mmol/L cysteine (dissolved in TES/SB) to each GCL activity tube and incubated at 37°C for 1 h before terminating the reaction by adding 50 μl of 200 mmol/L 5-sulfosalicylic acid (SSA). The GSH tube was incubated at 37°C at the same time and otherwise treated the same, except that the same amount of cysteine was added after instead of before termination. Both tubes were then vortexed and held on ice for at least 20 min, then centrifuged for 5 min at 3000 rcf in a Beckman tabletop centrifuge. Following centrifugation, 20 μl aliquots of supernatant from each tube were transferred to a 96-well plate designed for fluorescence detection. NDA derivatization solution (50 mmol/L Tris, pH 10.0, 0.5 mol/L NaOH, and 10 mmol/L NDA in dimethylsulfoxide (DMSO), v/v/v 1.4/0.2/0.2) were added to each well of this plate. The plate was covered to protect the wells from room light and allowed to incubate at room temperature for 30 min. The plate was read on a fluorescence plate reader with wavelengths set to 472 nm excitation/528 nm emission. The GCL activity was considered to be the fluorescence value of the GSH tube subtracted from the fluorescence value of the GCL tube.

### GSH/GSSG ratio assay

The GSH/GSSG ratio assay was performed by GSH/GSSG-Glo kit (Promega, WI) following provided protocol. Total glutathione (GSH+GSSG) and GSSG was quantified with standard curve, and GSH/GSSG ratio was calculated accordingly.

### G6PD activity assay

Cells were lysed ultrasonically on ice in 0.1 mole/L Tris and 0.5 mmole/L EDTA, pH 8.0. To aliquots of lysate containing equal amounts of protein, 10 mmole/L MgCl_2_ and 0.25 mmole/L NADP^+^ were added. The reaction was started at 37°C by adding 0.6 mmole/L glucose 6-phosphate and absorbance was measured in a spectrophotometer. G6PD activity was measured as the increase in absorbance/min at 340 nm [36]. The enzymatic activity was expressed as milliOD/min/μg protein.

### Statistical analysis

Statistical significance was established by student’s t test for single comparisons, or by ANOVA followed by the Tukey test or the LSD test for multiple comparison analysis.

## Results

### LM increased expression of mitochondrial electron transfer chain complexes

**Fig 1:**
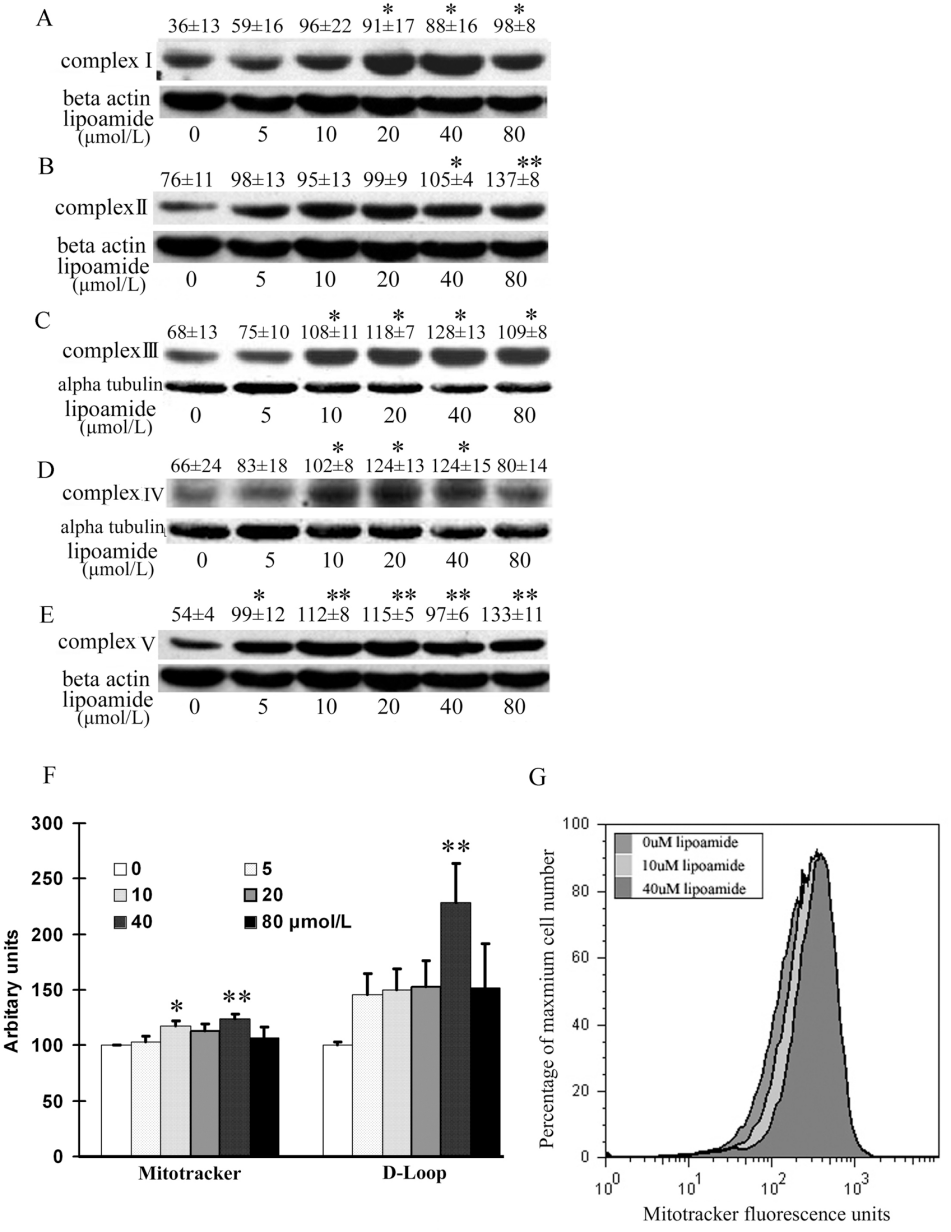
LM increased ETC complex I, II, III, IV and V protein expression (A-E), mitochondrial DNA copy number (F) and viable mitochondria (G). ARPE-19 cells were treated with the indicated concentrations of LM for 48 h, and then subunits expression of the complexes were detected by western blot. The subunits tested were 39 KD, 70 KD, 51.6 KD, 57 KD and 56.6 KD for complexes I to V (A to E), respectively. The images are representative; the quantitative results are from optical density analysis of images of three independent experiments. For complexes I, II and V, the loading control was β-actin; for complexes III and IV, it was α-tubulin instead. Results are the ratios of the complex densities to those of β-actin or α-tubulin. Values are means ± SEM. Differences were evaluated statistically with student’s t test. * p<0.05, and **p<0.01 vs. untreated control (0 μmol/L). **(F)** LM increased viable mitochondria and mitochondrial DNA copy numbers. ARPE-19 cells were treated with the indicated concentrations of LM for 48 h. For viable mitochondria measurement, cells were stained with Mitotracker Green. Fluorescence values read by flow cytometry were considered as estimates of viable mitochondria. Results are in arbitrary units normalized by setting the fluorescence of untreated (0 μmol/L LM) cells to 100. Values are means ± SEM from three independent experiments, each performed on three samples at each concentration. For mitochondrial DNA copy number measurement, real-time PCR was employed for assaying the D-LOOP region of mitochondrial DNA. The results shown are ratios of D-LOOP to 18S rDNA. Results are in arbitrary units normalized by setting the ratio of untreated (0 μmol/L LM) cells to 100. Values are means ± SEM from four independent experiments. Statistical significance of differences was established by student’s t test. * p<0.05, vs. 0 μmol/L treatment and **p<0.01 vs. untreated control (0 μmol/L) **(G)** A representative flow cytometry histogram was created with Flow Jo, Ver. 4.87 software. The fluorescence curves of 0, 10 and 40 μmol/L LM treatments were right-shifted with respect to the 0 μmol/L curve.

The electron transfer chain complexes located on the inner mitochondrial membrane are essential for mitochondrial function. We tested their protein expression after 48 hours of LM treatment, and found that LM stimulated the protein expression of all five complexes. For complex I, except at the low LM doses of 5 and 10 μmol/L, all other concentrations (20, 40 and 80 μmol/L) increased protein expression significantly (Fig 1A); for complex II, both 40 and 80 μmol/L increased the protein expression significantly (Fig 1B); for complex III, 10, 20, 40 and 80 μmol/L increased the protein expression significantly (Fig 1C); for complex IV, 10, 20 and 40 μmol/L increased the protein expression significantly (Fig 1D), and for complex V, all concentrations (5, 10, 20, 40 and 80 μmol/L) increased the protein expression significantly (Fig 1E).

### LM increased viable mitochondria mass

Treating ARPE-19 cells for 48 hours with different concentrations of LM, showed an increase in the number of viable mitochondria. The fluorescence unit of Mitotracker, a widely used stain for mitochondrial abundance, significantly increased by 23% at 40 μmol/L LM treatment, compared with control (Fig 1F and 1G). The mitochondrial DNA copy number, another marker of mitochondrial mass, was examined by comparing mitochondrial D-LOOP DNA and nuclear 18S rDNA. The result showed that LM treatment at 40 μmol/L, had a 2.2-fold increase in mitochondrial DNA copies, compared with control (Fig 1F).

### LM increased expression of mitochondrial biogenesis-related transcription factors

**Fig 2:**
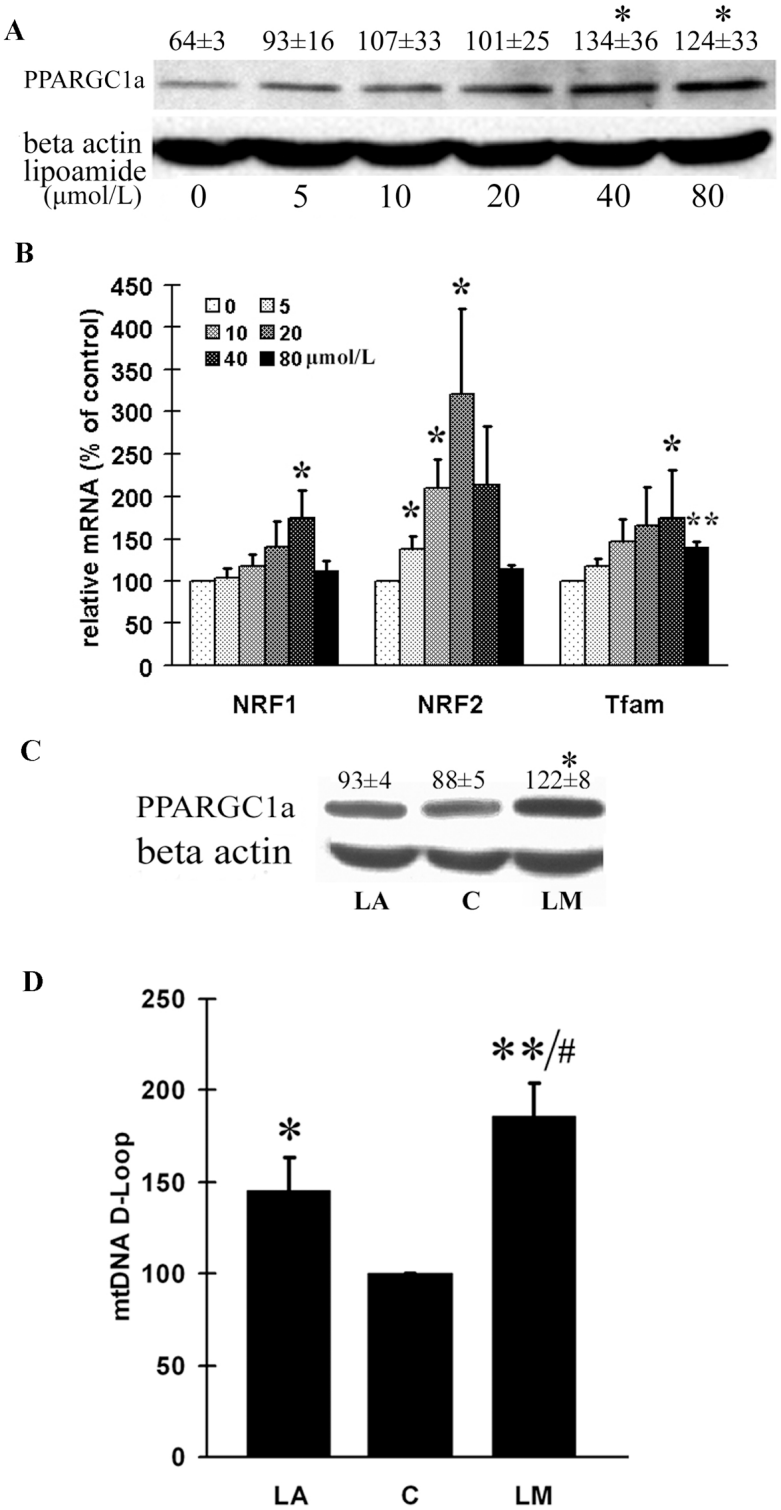
LM stimulated the expression of PPARGC1a, NRF-1, NRF-2 and Tfam, and LM was more potent than LA in inducing PPARGC1a expression and increasing mitochondrial DNA copy number. ARPE-19 cells were treated with the indicated concentrations of LM for 48 h. **(A)** A representative image PPARGC1a expression in protein was examined by western blot. Optical densities of four independent images were analyzed with Quantity One software, and results were calculated as ratios of PPARGC1a to β-actin densities. **(B)** NRF-1, NRF-2 and Tfam mRNA expression levels were examined by real-time RT-PCR. The results were calculated as ratios of NRF-1, NRF-2 or TFAm to 18srRNA levels. Values are means ± SEM from four independent experiments. **(C)** A representative western blot of PPARGC1a expression from four independent experiments. Optical densities of four independent images were analyzed with Quantity One software; results are expressed as ratios of PPARGC1a to β-actin. **(D)** Effects on mitochondrial DNA copy number. Real-time PCR was employed for assaying the D-LOOP region of mitochondrial DNA. The results are expressed as ratios of D-LOOP to 18S rDNA. Values are means ± SEM from four independent experiments. Results are in arbitrary units normalized by setting the ratio of control cells to 100. In both C and D, C stands for control, LM stands for 40 μmol/L LM treatment and LA stands for 40 μmol/L LA treatment. For both A and B, statistical significance was established by student’s t test. * p<0.05, and **p<0.01 vs. untreated control (0 μmol/L). For both C and D, statistical significance was established by one way ANOVA followed by the LSD test. * p<0.05, ** p<0.01 vs. untreated control (C, 0 μmol/L), and ^#^ p<0.05, vs. LA.

To confirm the effect of increasing mitochondrial mass, and to unveil the mechanism by which mitochondrial biogenesis is promoted by LM, we measured the expression of mitochondrial biogenesis transcription factors. PPARGC1a is the key transcriptional coactivator for inducing mitochondrial biogenesis. With LM treatment, the protein expression of PPARGC1a in ARPE-19 cells increased in a concentration-dependent manner, and both 40 and 80 μmol/L enhanced the protein expression significantly (Fig 2A). For the downstream transcription factors of PPARGC1a, we measured the mRNA expression of nuclear respiration factor 1 and 2 (*NRF1 and NRF2*), and *Tfam* with real time RT-PCR. LM significantly increased *NRF1* only at 40 μmol/L (by 74%). It increased the *NRF2* α-subunit expression at 5, 10, and 20 μmol/L, with the most effective concentration of 20 μmol/L causing a 3.2 fold increase. LM increased *Tfam* at 40 μmol/L (by 75%) and at 80 μmol/L (Fig 2B).

### LM was more potent than LA in inducing PPARGC1a expression and increasing mtDNA copy number

Since LA has been proved to be a mitochondrial biogenesis inducer [37], we compared the abilities of LM and LA in inducing mitochondrial biogenesis. Consistent with previous results, the key transcriptional coactivator PPARGC1a was significantly induced by LM treatment (40 μmol/L for 48 h), but not by LA at the same concentration (Fig 2C). For mitochondrial DNA, we found both LM and LA could significantly increase mitochondrial DNA copy number, but the effect of LM treatment was more significant than that of LA (Fig 2D).

### LM stimulated oxygen consumption, increased MMP and inhibited ROS production

**Fig 3:**
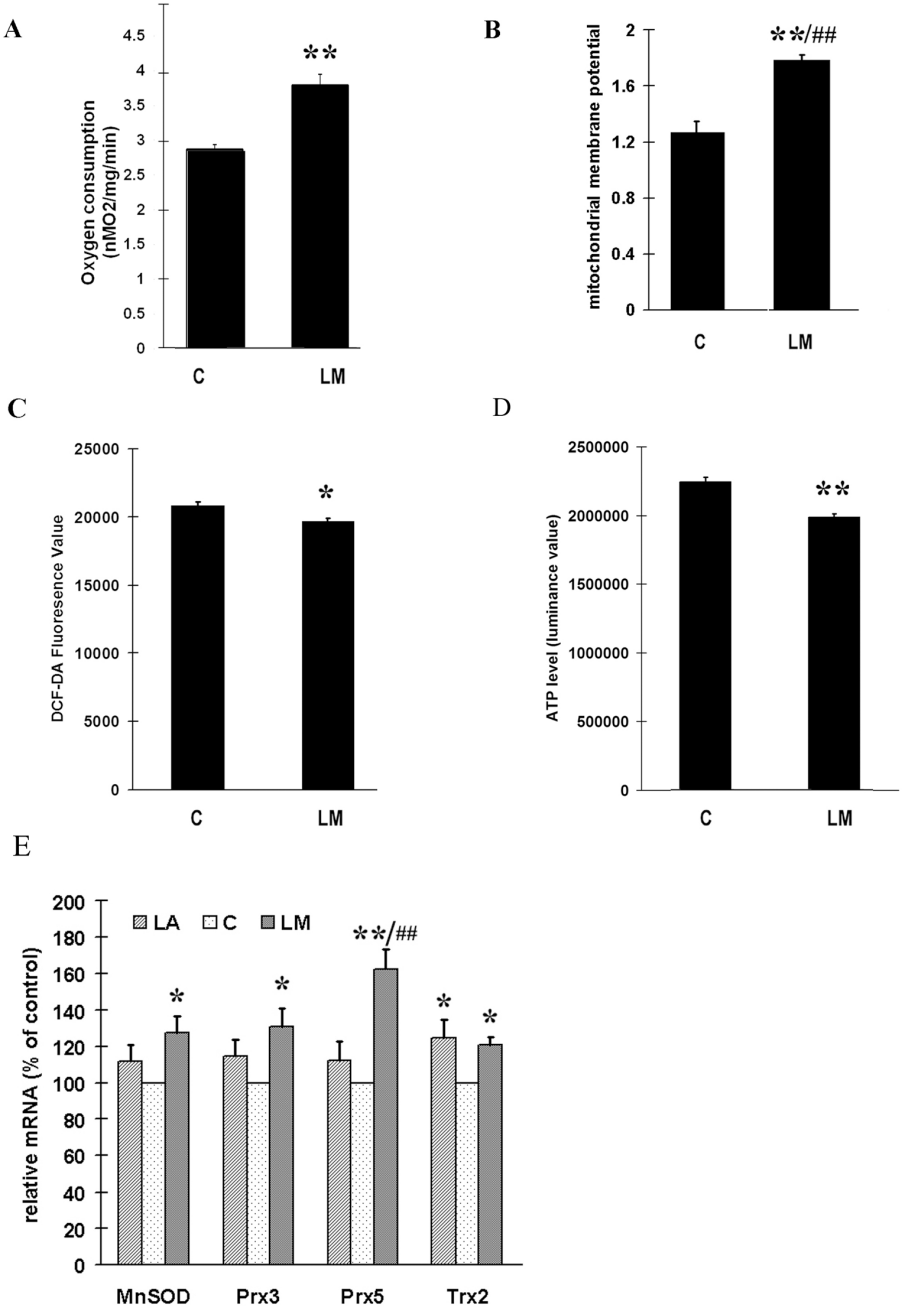
The effects of LM on oxygen consumption (A), mitochondrial membrane potential (MMP) (B), ROS production (C); cellular ATP level (D) and the expression of MnSOD,Trx2,Prx3,and Prx5 (E). ARPE-19 cells were treated with 40 μmol/L LM for 48 hours; then the following assays were carried out immediately. **(A)** LM promoted oxygen consumption. Results are expressed as the rate of oxygen consumption, with media without cells used as a blank. Values are means ± SEM from three independent experiments; three parallel measurements were used for each sample in every experiment. **(B)** LM treatment increased MMP as determined by JC-1 staining. Values are means ± SEM of the ratio of fluorescence at 590 nm to 530 nm from three independent experiments; 4 parallel wells for each group were used in each experiment. **(C)** LM treatment decreased ROS production examined by DCF-DA staining. Values are means ± SEM of 8 parallel wells of a representative experiment, from four independent experiments each showing similar trends. (D) LM treatment decreased cellular ATP level. Values are means ± SEM from 3 independent experiments. (E) Expression of MnSOD,Trx2,Prx3,and Prx5. ARPE-19 cells were treated with 40 μmol/L LM or LA for 48 h; then RNA was isolated and reverse-transcribed to cDNA. Real time PCR was employed to measure expression levels of the indicated genes. The results (from 5 independent experiments) are expression ratios of the target genes to 18SrRNA, and are normalized to control (control = 100). C stands for control, LM stands for 40 μmol/L LM treatment and LA stands for 40 μmol/L LA treatment. Statistical significance was established by one way ANOVA followed by the Tukey test (A, B, C, D) or LSD test (E). * p<0.05, ** p<0.01 vs. untreated control (0 μmol/L); ^#^p<0.05, ^##^ p<0.01 vs. LA.

AS the above results strongly indicate the fact that LM stimulates mitochondrial biogenesis, we further evaluated mitochondrial function by measuring oxygen consumption and mitochondrial membrane potential (MMP) in ARPE-19 cells. LM treatment (40 μmol/L for 48 h) caused a 34% increase in oxygen consumption (Fig 3A), and a significant increase (40%) in MMP (Fig 3B) compared with untreated control. We were also concerned whether the higher oxygen consumption and MMP might lead to higher ROS production. However, LM treatment did not cause an increase—on the contrary, it showed a small but still statistically significant inhibition of ROS (Fig 3C). At the same time, we surprisingly found the cellular ATP level was decreased by 10% by LM treatment (Fig 3D), which seemed to be conflict with higher oxygen consumption. This unexpected phenomenon was addressed in the latter part of this article.

### LM increased mRNA expression of MnSOD, Trx2, Prx3 and Prx5

Overexpression of PPARGC1a can upregulate cell mitochondrial antioxidant protein expression [31]. As LM increased PPARGC1a expression in ARPE-19 cells and antioxidant capacity of ARPE-19 cells is crucial in our study, we tested the effect of LM, in comparison with LA, on the mRNA expression of *MnSOD*, *Trx2*, *Prx3*, and *Prx5* with real time RT-PCR. As shown in Fig 3E, LM treatment (40 μmol/L for 48 h) significantly increased the mRNA expression of *MnSOD*, *Trx2*, *Prx3*, and *Prx5*, whereas LA treatment at the same concentration during the same period only significantly increased *Trx2* mRNA expression.

### LM promoted Nrf2 expression in both nuclear and cytosolic protein fractions

**Fig 4:**
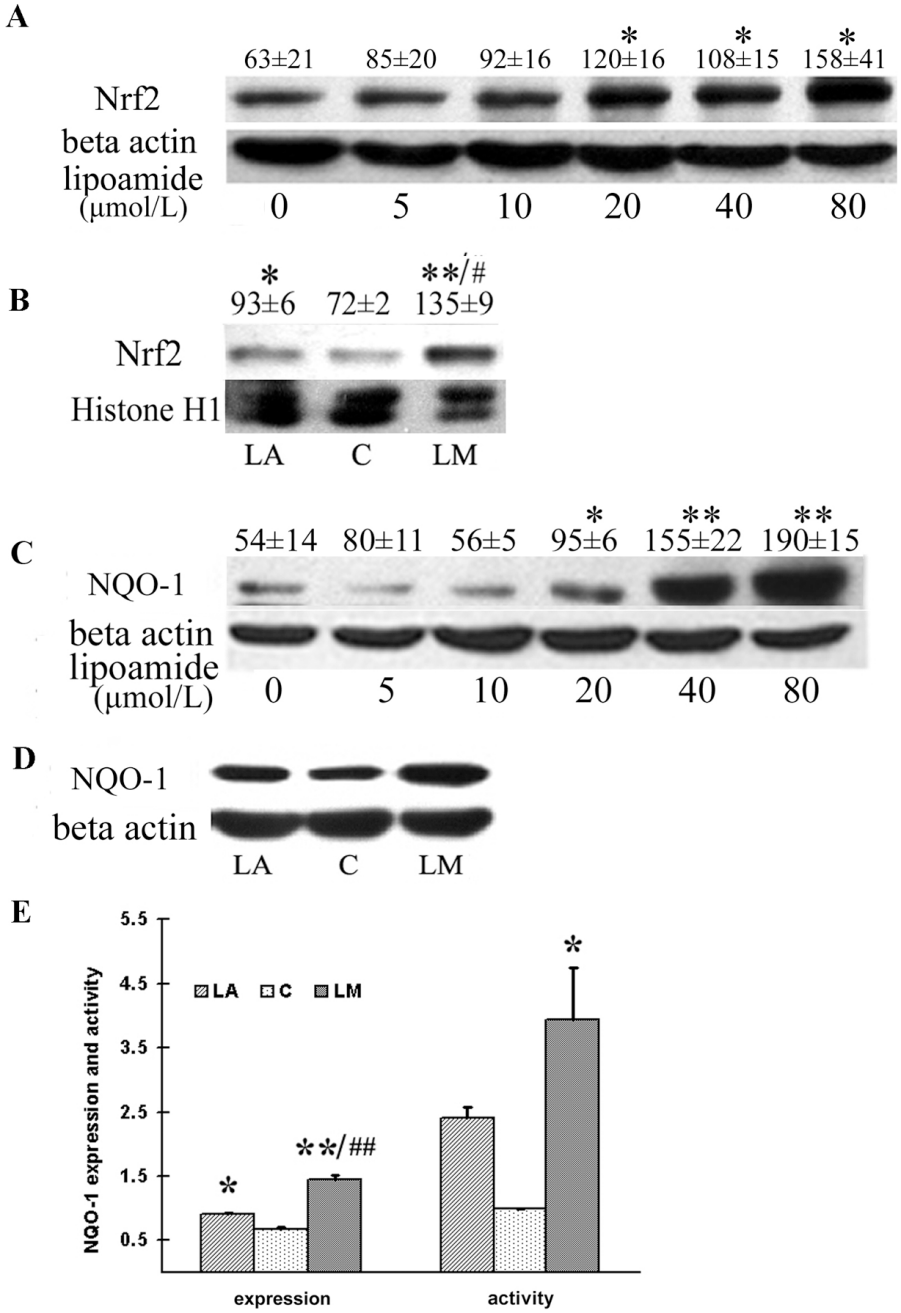
LM treatment increased Nrf2 expression in both total and nuclear protein fraction, and increased expression and activity of NQO-1. ARPE-19 cells were treated with the indicated concentrations of LM for 48 h, or 40 μmol/L of LA or LM if concentrations not indicated. **(A)** A representative image of Nrf2 expression in total protein detected by western blot. Optical densities were analyzed with Quantity One software, and results are expressed as ratios of Nrf2 to β-actin in arbitrary units. **(B)** A representative image of Nrf2 expression in nuclear protein. Quantitative analysis of Nrf2 expression in nuclear protein was quantified in the same way as for Nrf2 expression in total protein. **(C)** A representative image of NQO-1 expression detected by western blot. Quantitative analysis of NQO-1 expression in total protein was performed in the same wa with Nrf2.. **(D)** A representative image of NQO-1 expression. **(E)** Quantitative analysis of NQO-1 expression and activity. All values are means ± SEM of four independent experiments. Statistical significance was established by one way ANOVA followed by the Tukey test. *p<0.05, and **p<0.01 vs. control, and ^#^p<0.05, ^##^p<0.01 vs. LA treatment.

We have previously shown that LM pretreatment protects ARPE-19 cells from the acrolein-induced oxidative stress. Then we asked whether LM could directly react with free radicals. However, in the DPPH assay, we found neither LA nor LM directly scavenge DPPH free radical when compared with ascorbic acid (S1 Fig). These results implied that LA and LM may boost cellular antioxidant ability acting as indirect antioxidant. Nrf2 (nuclear factor erythroid 2-related factor 2) is the key transcription factor for regulating antioxidant response element- (ARE)-based gene expression of phase II detoxifying enzymes. We demonstrated that LM treatment at 20, 40 and 80 μmol/L promoted Nrf2 expression (Fig 4A). LM treated at 40μmol/L was more potent than LA at the same concentration in inducing Nrf2 nuclear translocation (Fig 4B). As we concerned that the cell density may affect the effect of LM and LA in activating Nrf2, we also used fully confluent cells to verify our data. The results (S3 Fig) showed that nuclear Nrf2 protein levels in fully confluent cells were also increased by LM and LA treatment.

### LM increased expression and/or enzymatic activities of phase II antioxidant enzyme (NQO-1, GST, Cu/ZnSOD and G6PD)

NQO-1 is one of the phase II antioxidant enzyme that is regulated by Nrf2. We examined the expression and activity of NQO-1. Consistent with findings on Nrf2, the protein expression of NQO-1 was increased by LM treatment at 20, 40 and 80 μmol/L(Fig 4C). We also compared the effects of LM and LA at 40 μmol/L on NQO-1 expression and activity, and found that both LM and LA could significantly stimulate NQO-1 expression and activity (Fig 4D and 4E); LM showed a more potent effect than LA, but the difference was not statistically significant.

**Fig 5:**
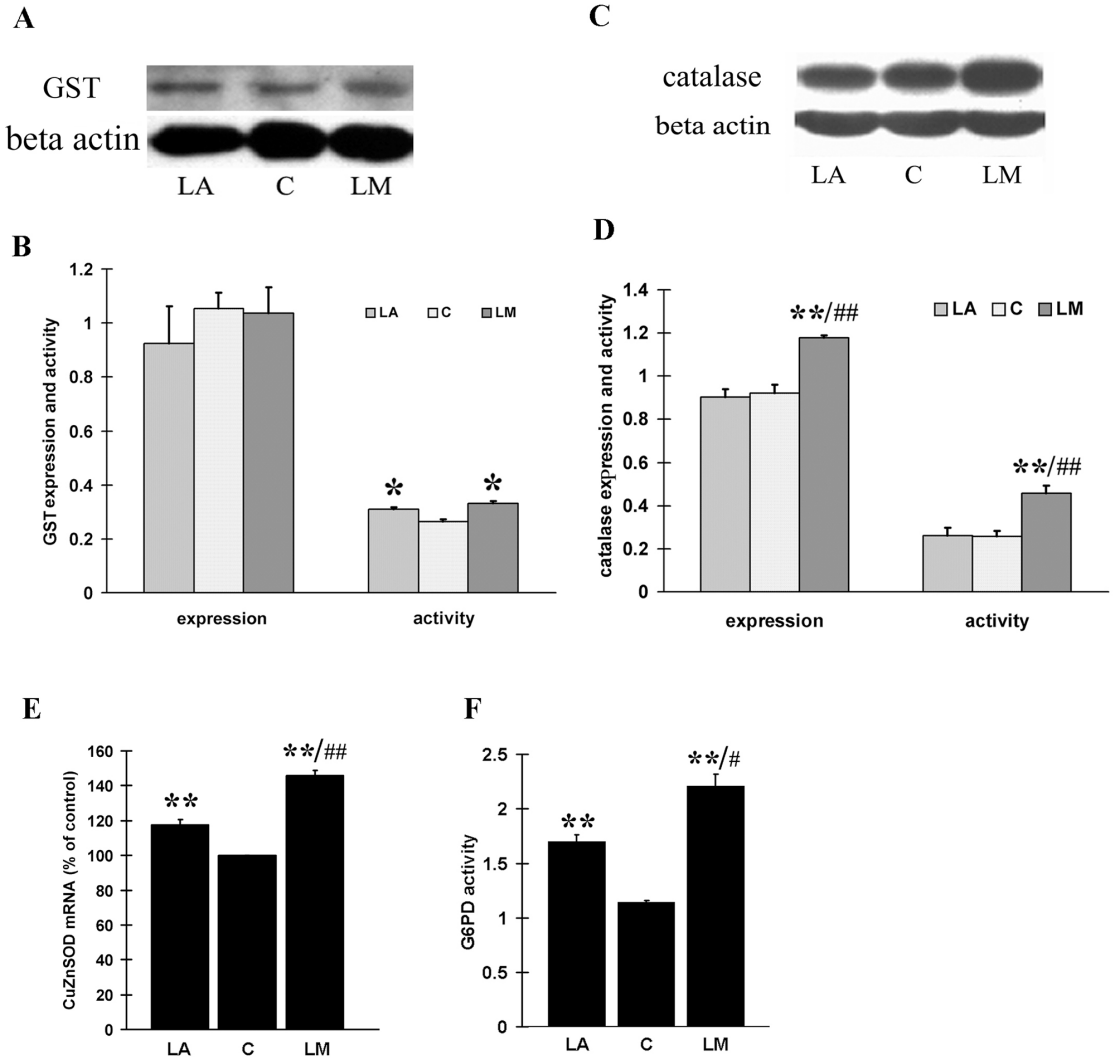
The effects of LM and LA on expression and activity of GST, catalase, Cu/ZnSOD and G6PD. ARPE-19 cells were treated with 40 μmol/L LM or LA for 48 h. **(A)** A representative image of total GST expression detected by western blot. **(B)** Quantitative analysis of GST expression (4 independent experiments) and activity (5 independent experiments). **(C)** A representative image of catalase expression detected by western blot. **(D)** Quantitative analysis of catalase expression (4 independent experiments) and activity (3 independent experiments). The results are expressed in arbitrary units and each experiment was performed in duplicate. **(E)** Transcriptional expression of Cu/ZnSOD. Real time RT-PCR was employed to measure expression levels of Cu/ZnSOD. Results (from 5 independent experiments) are expressed as ratios of Cu/ZnSOD to 18SrRNA. **(F)** G6PD activity was measured as described in Methods. Values are means ± SEM of three independent experiments. All statistical significance were established by one way ANOVA followed by the Tukey test. * p<0.05, **p<0.01 vs. untreated controls (0 μmol/L), ^#^p<0.05 vs. LA, and ^##^p<0.01 vs. LA.

GST is another important phase II enzyme. The activity of total GST was increased by 19% and 27% by treatment with 40 μmol/L LM and LA, respectively (Fig 5B). However, protein expression of total GST was not increased by either LM or LA treatment (Fig 5A and 5B).

Catalase and Cu/ZnSOD are important endogenous antioxidant enzymes, and both were regulated by Nrf2. We tested the effect of LM and compared its effect with that of LA. LM treatment (40 μmol/L, 48 h) caused a significant increase in both expression and activity of catalase, but LA treatment (40 μmol/L, 48 h) didn’t show any obvious effects on either the expression or activity of catalase (Fig 5C and 5D). We also found both LA and LM could significantly increase the Cu/ZnSOD mRNA level, but the effect of LM was not potent (Fig 5E). Besides, glucose-6-phosphate dehydrogenase (G6PD), the key enzyme in NADPH generation, is also an Nrf2-regulated enzyme, and we found its activity exibited a more than two-fold increase after LM treatment, while only 50% increase after LA treatment (Fig 5F).

### LM increased GCL expression, enzymatic activity, GSH levels and GSH/GSSG ratio

**Fig 6:**
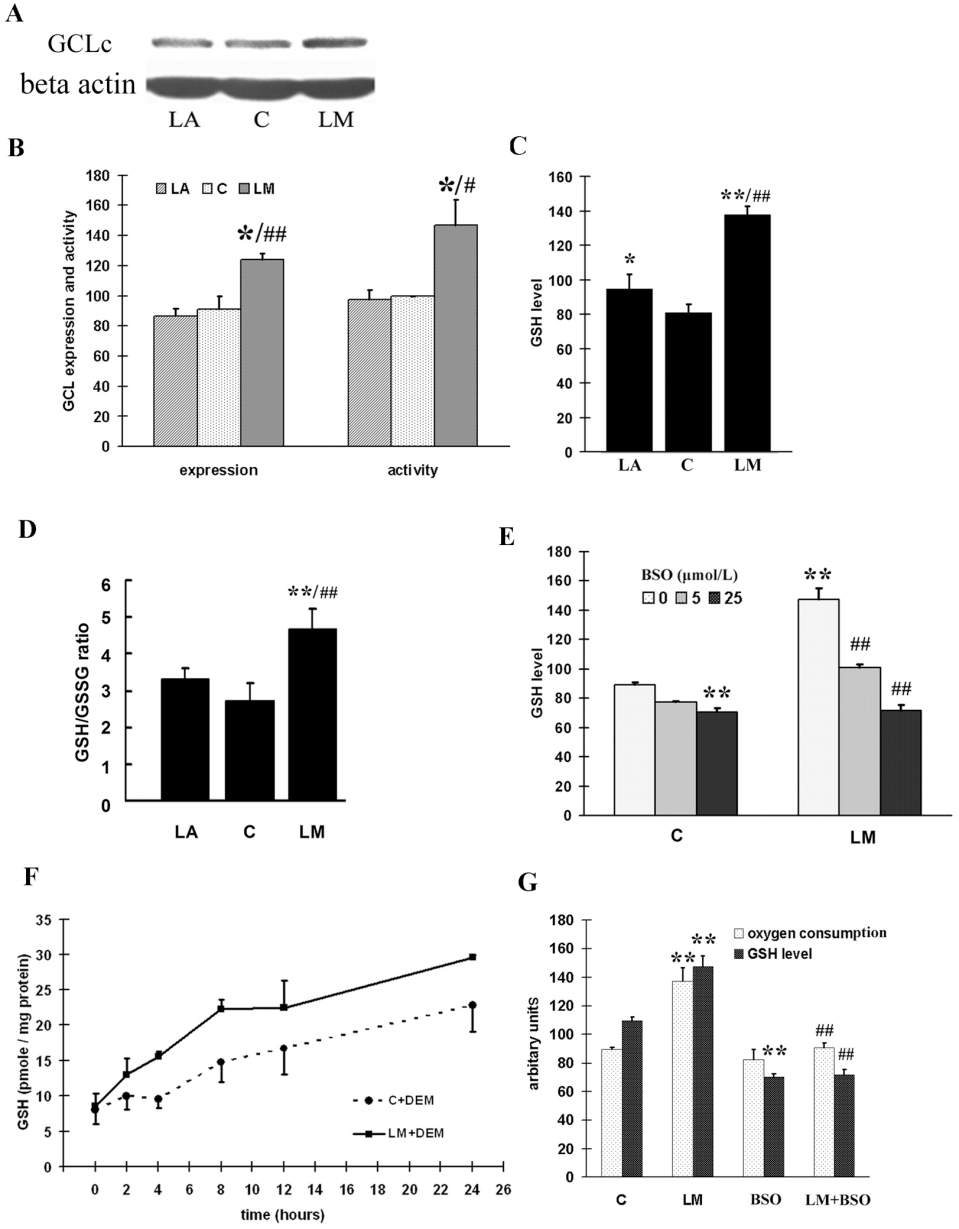
GSH levels was increased in LM treated ARPE-19 cells, and enhanced oxygen consumption was related to *de novo* GSH synthesis. **(A)** A representative image of GCLc expression detected by western blot. **(B)** Quantitative analysis of GCLc expression (4 independent experiments) and GCL activity (3 independent experiments). **(C)** Quantitative analysis of GSH level. Averages of four independent experiments are displayed in arbitrary units. Values are means ± SEM. **(D)** Quantitative analysis of GSH/GSSG ratio Total glutathione and oxidative form glutathione (GSSG) was measured as described in method, GSH/GSSG ratio was caculated. Values are means ± SEM from three independent experiments. **(E)** LM increases GSH levels by GCL activation. ARPE-19 cells were treated with or without 40 μmol/L LM, and 5 or 25 μmol/L BSO was added to the media at the same time; after 48 h, GSH levels were measured spectrometrically as described in Materials and Methods. The results in arbitary units are averages of three independent experiments. Values are means ± SEM. **(F)** LM treatment improved recovery of cellular GSH levels with time. ARPE-19 cells were treated with or without 40 μmol/L LM for 48 h, and then exposed to media containing 2 mmol/L diethyl maleate (DEM) for 90 min to deplete cellular GSH; the recovery of GSH was examined over time. LM+DEM recovery was significantly higher than C+DEM. The results are shown as means ± SEM from four independent experiments. Statistical significance was established by two way ANOVA followed by the LSD test. **(G)** Effect of LM on BSO-induced decrease in oxygen consumption and GSH levels. ARPE-19 cells were treated with or without LM (40 μmol/L) or BSO (25 μmol/L) for 48 h, then the GSH level and oxygen consumption were examined as described in Materials and Methods. Values are means ± SEM of three independent experiments. For A, B, C, D, E and G, statistical significance was established by one way ANOVA followed by the Tukey test. In A, B, C and D* p<0.05, **p<0.01 vs. untreated controls (0 μmol/L), ^#^p<0.05 vs. LA, and ^##^p<0.01 vs. LA. In E and G, **p<0.01 vs. untreated controls, and ^##^p<0.01 vs. LM treatment

GCL, which ligates L-glutamate and L-cysteine, is the key enzyme in GSH *de novo* synthesis. We examined the expression of GCLc, the catalytic subunit of GCL, and the activity of GCL. With 40 μmol/L LM treatment, GCLc expression was significantly increased (Fig 6A and 6B). Similarly, GCL activity was also increased significantly (by 45%) by LM treatment (Fig 6B). LA treatment showed no significant effect on either the expression of GCLc or the activity of GCL (Fig 6A and 6B).

GSH is the final product of GCL catalysis and is considered to be the most important low weight antioxidant molecule, as GSH levels usually determines the redox status of the cell. LM treatment induced a 1.7 fold increase in the GSH level compared with control; LA treatment also induced a significant but much smaller increase (20%) compared with control, and the effect of LM was significantly greater than LA (Fig 6C). GSH/GSSG ratio, the indicator of cellular redox status, increased by 70% with LM treatment; while LA treatment doesn’t significantly increase the ratio (Fig 6D).

### Enhanced mitochondrial function is to sustain high cellular GSH levels with LM treatment

To find out whether the increase of GSH level was due to GCL activation, the GCL inhibitor BSO was used to block *de novo* GSH generation; and we found that the increased GSH level caused by LM treatment was very sensitive to BSO inhibition (Fig 6E). It’s reported that the depletion of GSH by diethyl maleate cannot be replenished if GCL is inhibited [38]. We found that the GSH level recovered faster in the presence of LM compared to control after we depleted cellular GSH with diethyl maleate treatment (Fig 6F). These results demonstrated that LM treatment increased *de novo* GSH synthesis through GCL activation.

As we mentioned above, LM treatment increased oxygen consumption by about 40% compared to control (Figs 3A and 6G). However, intracellular ATP levels did not increase but declined 10% (Fig 3D). We speculated that the LM-induced energy expenditure may have been used for *de novo* GSH synthesis. We then treated the cells with BSO to inhibit GCL activity. Under these conditions, in both LM-treated and LM-untreated cells, GSH levels decreased to the same level, about 60% of control. Oxygen consumption also decreased to the same level (Fig 6G). The mitochondrial membrane potential and ROS level had no significant change with BSO treatment (S2 Fig), which indicated the decrease of oxygen consumption after BSO treatment was not due to GSH decline caused oxidative stress. These results indicate that the additional oxygen consumption was used to generate *de novo* GSH.

## Discussion

Accumulated evidences suggest oxidative damage and mitochondrial dysfunction are the major causes of aging and age-related degenerative diseases and that improving mitochondrial function and inhibiting oxidative damage are effective strategies for delaying aging and preventing age-related diseases [7, 39]. LA has been proven to be a mitochondrial antioxidant nutrient and an inducer of phase II antioxidant enzymes [20]. More recently, LA has been shown to promote mitochondrial biogenesis and function in 3T3-L1 adipocytes [11] and in SK-N-MC neuroblastoma cells [40]. LM is the amide derivative of LA, and was found to be more potent than LA in protecting ARPE-19 cells from acrolein-induced oxidative damage and mitochondrial dysfunction [22]. In this study, we demonstrated that LM stimulated mitochondrial biogenesis by increasing PPARGC1a expression and induced Nrf2-regulated phase II antioxidant enzymes in ARPE-19 cells; meanwhile, we showed LM was more potent than LA in the above-mentioned capabilities.

LM acts as a stimulator of mitochondrial biogenesis and simultaneously as an inducer of phase II antioxidant enzyme. It suggests that mitochondrial biogenesis and phase II antioxidant systems are closely related or coupled. The relation between mitochondrial biogenesis and ROS production has been well debated [41]. Mitochondrial biogenesis can lead to enhancement of both intracellular and mitochondrial antioxidant system. PPARGC1a, the most important transcriptional coactivator in inducing mitochondrial biogenesis, also affects the cellular antioxidant system. Liang et al. [3] found that overexpression of PPARGC1a promoted the antioxidant ability of 3T3 fibroblasts; Valle et al. [31] found the expression of PPARGC1a promoted the expression of mitochondrial antioxidant enzymes. St-Pierre et al. [42] proved PPARGC1a is required for the expression of antioxidant enzymes, and PPARGC1a expression enhances antioxidant ability and reduces ROS in neurons. In our previous study, we found LM pretreatment protected ARPE-19 cells from acrolein-induced oxidant stress and mitochondrial dysfunction. In this study, we further showed that LM-induced increase in PPARGC1a expression led to an increase in mitochondrial biogenesis and function, and also enhanced gene expression of mitochondrial antioxidant enzymes, including SOD, Trx2, Prx3 and Prx5 (Fig 3E). Moreover, we found that increased mitochondrial mass was accompanied by an increase in MMP (Fig 3B), mitochondrial complex expression (Fig 1), oxygen consumption and a decrease in ROS production (Fig 3A and 3C).

However, the small but significant decline (10%) in ATP was unexpected (Fig 3D). We propose that the ATP decrease may be related to the increased antioxidant levels, especially to the increased generation of GSH. There may be an energy link between the mitochondrial biogenesis stimulation and phase II enzyme induction. The *de novo* generation of the most important phase II enzyme product—GSH, is highly related to energy supply, because generation of a new GSH molecule requires consumption of two ATP molecules [43, 44]. With a concentration in the micromolar scale, GSH is abundant in the cell. LM increased GSH levels by 50–70% in ARPE-19 cells(Fig 6C). This must require a large amount of energy, which may be compensated for by the increased mitochondrial mass and improved mitochondrial function. Pantothenic acid is reported to increase intracellular GSH levels by promoting ATP production [45] and mitochondrial uncouplers or ATP synthase inhibitors can lower intracellular GSH levels [46]. We showed that LM-treated cells tended to maintain higher GSH level increased GSH generation was related to increased oxygen consumption, and blocking GSH synthesis recovered the oxygen consumption to near normal level (Fig 6G). These results imply that improvement of mitochondrial biogenesis or mitochondria function is necessary for generating sufficient ATP to maintain high GSH level.

NADPH-consuming antioxidant enzymes (NQO-1, and enzymes involved in GSH recycling, etc.) may also be affected by energy supply. Glucose-6-phosphate dehydrogenase (G6PD), the key enzyme in NADPH generation, and the first rate-limiting enzyme in pentose-phosphate pathway, is also an Nrf2-regulated enzyme, and we found its activity had a more than two-fold increase after LM treatment (Fig 5F). The increased requirement for NADPH and greater G6PD activation should shunt more glucose into the pentose-phosphate pathway, which may inhibit glycolysis and make the cell more dependent on mitochondrial function. That provides a possible explanation linking induction of phase II antioxidant enzymes and stimulation of mitochondrial biogenesis and function. However, whether induction of other phase II antioxidant enzymes correlate with mitochondrial biogenesis and function for the sake of ATP supplementation, warrants further investigation.

In conclusion, we found LM treatment stimulated mitochondrial biogenesis and function and induced phase II enzyme expression in ARPE-19 cells. Enhanced mitochondrial biogenesis, improved mitochondrial function and strengthened antioxidant defense might account for the protecting mechanism of LM against acrolein-induced oxidant damage and mitochondrial dysfunction.

## Supporting Information

S1 Fig LA or LM doesn’t directly scavenge DPPH free radical. (A) Doses of 40 μM ascorbic acid, lipoic acid or lipoic amide were added to 60 μM DPPH free radical. Absorbance was measured after incubation for 90 minutes at 37°C following addition (One-way ANOVA followed by Tukey’s test, n = 8 per group); (B) Different doses of lipoic acid, lipoamide or ascorbic acid were added to 50 μM DPPH, and absorbance was measured after incubation for 30 minutes at 37°C following addition (n = 4 per group).
(TIF)

S2 Fig BSO treatment doesn’t change mitochondrial membrane potential or ROS production in ARPE-19 cells. ARPE-19 cells were treated with indicated concentrations of BSO for 48 hours, and Jc-1 assays and H_2_DCFDA staining assays were performed as described in the method. (A) Mitochondrial membrane potential measured by Jc-1 staining. Values are means ± SEM of the ratio of fluorescence at 590 nm to 530 nm from three independent experiments; 4 parallel wells for each group were used in each experiment. (B) ROS production examined by H_2_DCFDA staining. Values are means ± SEM of 4 parallel wells of a representative experiment, from four independent experiments each showing similar trends.
(TIF)

S3 Fig LA or LM treatment increases nuclear Nrf2 protein in confluent ARPE-19 cells. ARPE-19 cells were seeded at confluent density in 100 mm cell culture dishes, and maintained for two days; then the cells were treated with indicated concentrations of LM for 48 h (A), or 40 μmol/L of LA or LM for 48 h (B). Nuclear protein were extracted, and western blots were performed as described in method. (A) and (B) are representative images from three independent experiments which have same trends.
(TIF)
