# Supplementary figures and images for: Lipoamide Acts as an Indirect Antioxidant by Simultaneously Stimulating Mitochondrial Biogenesis and Phase II Antioxidant Enzyme Systems in ARPE-19 Cells
Source: PLoS One. 2015 Jun 1;10(6):e0128502. doi: 10.1371/journal.pone.0128502 (PMC4452644; doi:10.1371/journal.pone.0128502)

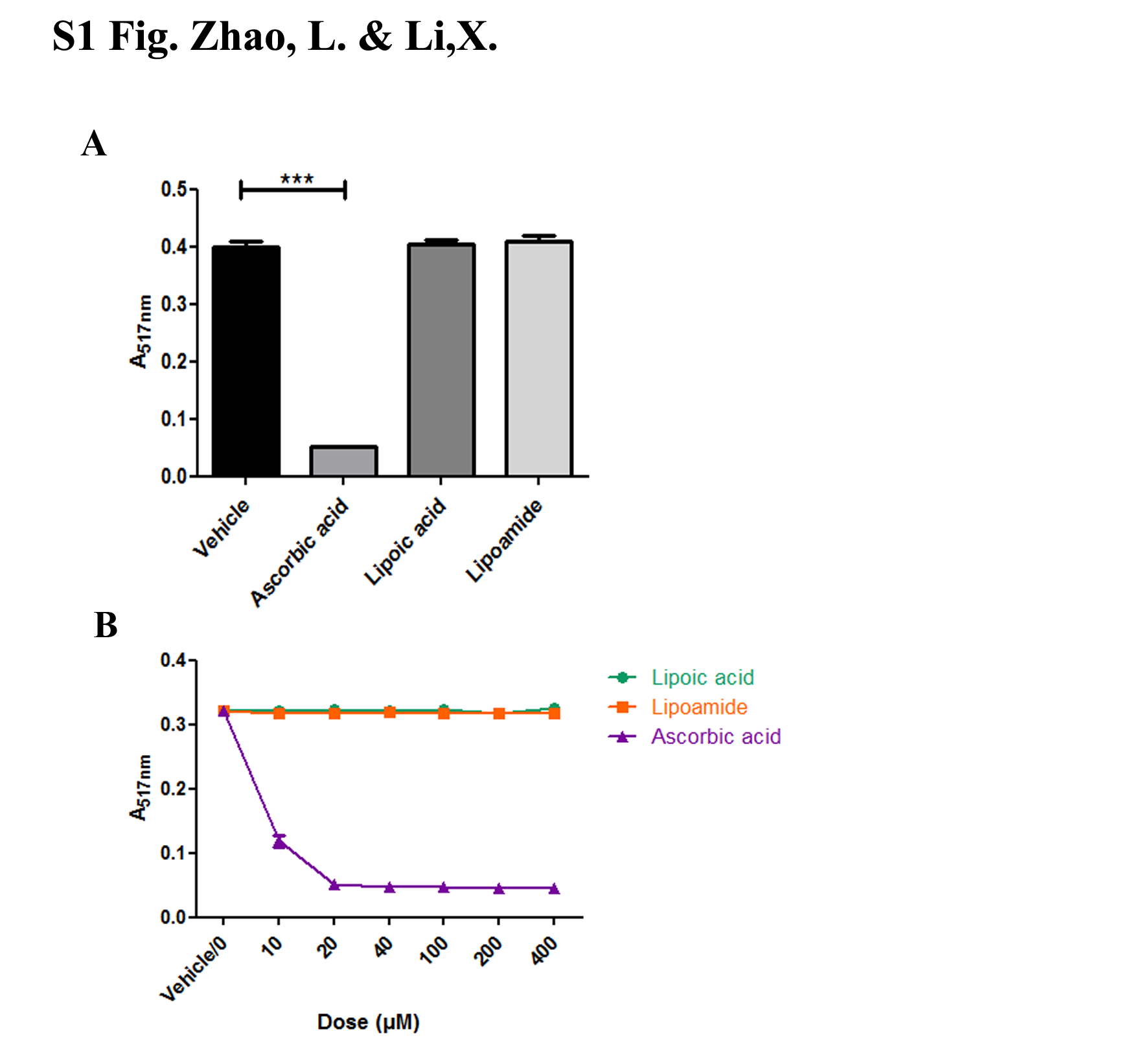

Supplement: S1 Fig — (A) Doses of 40 μM ascorbic acid, lipoic acid or lipoic amide were added to 60 μM DPPH free radical. Absorbance was measured after incubation for 90 minutes at 37°C following addition (One-way ANOVA followed by Tukey’s test, n = 8 per group); (B) Different doses of lipoic acid, lipoamide or ascorbic acid were added to 50 μM DPPH, and absorbance was measured after incubation for 30 minutes at 37°C following addition (n = 4 per group). (TIF) [file pone.0128502.s001.tif]

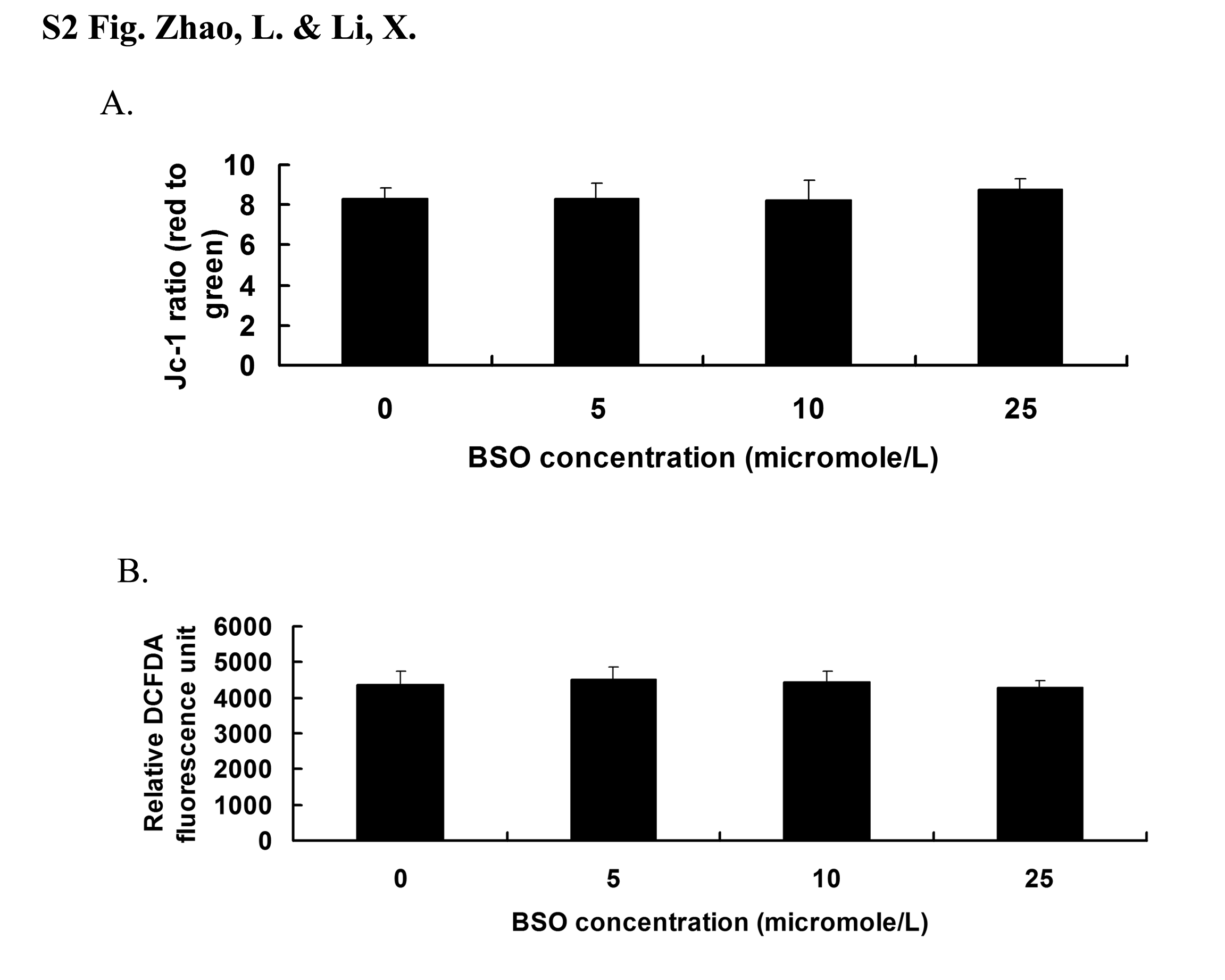

Supplement: S2 Fig — ARPE-19 cells were treated with indicated concentrations of BSO for 48 hours, and Jc-1 assays and H2DCFDA staining assays were performed as described in the method. (A) Mitochondrial membrane potential measured by Jc-1 staining. Values are means ± SEM of the ratio of fluorescence at 590 nm to 530 nm from three independent experiments; 4 parallel wells for each group were used in each experiment. (B) ROS production examined by H2DCFDA staining. Values are means ± SEM of 4 parallel wells of a representative experiment, from four independent experiments each showing similar trends. (TIF) [file pone.0128502.s002.tif]

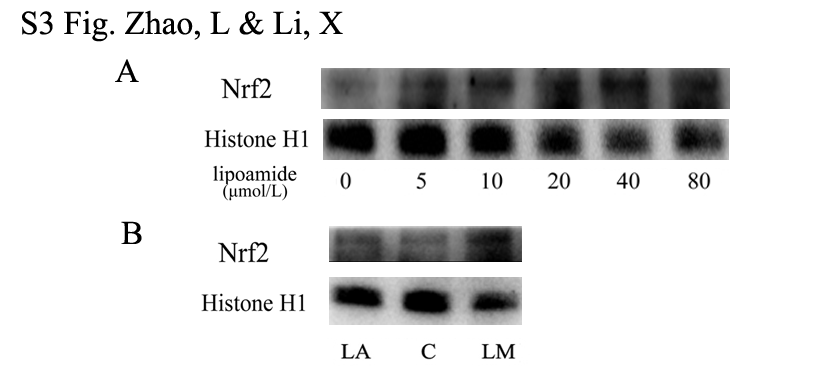

Supplement: S3 Fig — ARPE-19 cells were seeded at confluent density in 100 mm cell culture dishes, and maintained for two days; then the cells were treated with indicated concentrations of LM for 48 h (A), or 40 μmol/L of LA or LM for 48 h (B). Nuclear protein were extracted, and western blots were performed as described in method. (A) and (B) are representative images from three independent experiments which have same trends. (TIF) [file pone.0128502.s003.tif]
